# Determinants of CD4 cell count change and time-to default from HAART; a comparison of separate and joint models

**DOI:** 10.1186/s12879-018-3108-7

**Published:** 2018-04-27

**Authors:** Awoke Seyoum Tegegne, Principal Ndlovu, Temesgen Zewotir

**Affiliations:** 1 Department of statistics, Bahir Dar University, Bahir Dar, Ethiopia; 2 Department of Statistics, Unisa, Pretoria, South Africa; 3 School of Mathematics, Statistics and Computer Science, University of Kwazulu Natal, Durban, South Africa

**Keywords:** CD4 cell count change, GLMM, Heterogeneity, Homogeneity, Joint model, Time to default

## Abstract

**Background:**

HIV has the most serious effects in Sub-Saharan African countries as compared to countries in other parts of the world. As part of these countries, Ethiopia has been affected significantly by the disease, and the burden of the disease has become worst in the Amhara Region, one of the eleven regions of the country. Being a defaulter or dropout of HIV patients from the treatment plays a significant role in treatment failure. The current research was conducted with the objective of comparing the performance of the joint and the separate modelling approaches in determining important factors that affect HIV patients’ longitudinal CD4 cell count change and time to default from treatment.

**Methods:**

Longitudinal data was obtained from the records of 792 HIV adult patients at Felege-Hiwot Teaching and Specialized Hospital in Ethiopia. Two alternative approaches, namely separate and joint modeling data analyses, were conducted in the current study. Joint modeling was conducted for an analysis of the change of CD4 cell count and the time to default in the treatment. In the joint model, a generalized linear mixed effects model and Weibul survival sub-models were combined together for the repetitive measures of the CD4 cell count change and the number of follow-ups in which patients wait in the treatment. Finally, the two models were linked through their shared unobserved random effects using a shared parameter model.

**Results:**

Both separate and joint modeling approach revealed a consistent result. However, the joint modeling approach was more parsimonious and fitted the given data well as compared to the separate one. Age, baseline CD4 cell count, marital status, sex, ownership of cell phone, adherence to HAART, disclosure of the disease and the number of follow-ups were important predictors for both the fluctuation of CD4 cell count and the time-to default from treatment. The inclusion of patient-specific variations in the analyses of the two outcomes improved the model significantly.

**Conclusion:**

Certain groups of patients were identified in the current investigation. The groups already identified had high fluctuation in the number of CD4 cell count and defaulted from HAART without any convincing reasons. Such patients need high intervention to adhere to the prescribed medication.

## Background

HIV is more prevalent in Sub-Saharan African countries like Ethiopia, and accounted for approximately 71% of world total of HIV infected people in 2013 [1, 2]. In Ethiopia the incidence rate was 1.5% [3, 4]. As other regions in Ethiopia, the Amhara region, the study area, is highly affected by the disease [5].

Highly Active Antiretroviral Therapy (HAART) is a life time treatment therapy given to HIV infected individuals. The therapy is given as a combination of different medication drugs based on the mechanism of treating the viruses [6], and the usual measure of success or failure of the therapy is the patient’s CD4 cell count. Other factors that can affect the CD4 cell count of patients on HAART are: age (aged patients have lower CD4 count responses to HAART [7–9]); sex (females have higher CD4 count responses to HAART [10]); and residential area (rural patients have lower CD4 cell count responses to HAART [11]). Some earlier scholars also showed that WHO HIV stages are a self-determining indicator for the variation or fluctuation of CD4 cell count change at the starting time of HAART [12]. Another study reported that there is a positive correlation between baseline CD4 cell count change and the CD4 cell count after the commencement of HAART [13, 14].

Earlier studies were conducted on the joint modeling of CD4 cell count change and time to default from the treatment considered that the CD4 cell count change as continuous variable [15] but the distribution of the CD4 count change, whatever it is, is discrete. This means the formal statistical results of [15] maybe invalid because of regarding discrete response CD4 cell count change as continuous. Furthermore, previous joint models were conventional linear mixed effect models with the assumption of constant within subject variance [16] a restrictive assumption which if violated by the data renders statistical analysis results invalid. The above mentioned shortcomings on what has been done on the joint modeling suggested considering the more flexible generalized linear mixed effect models in conjunction with parametric and semi parametric survival time models for the joint and separate modeling of CD4 cell count change and time to default from HAART to determine predictors of these patients’ responses. As far as we are aware of the literature, no other such investigation has been conducted.

## Methods

### Source of data

The longitudinal data used in this study consists of records of 792 HIV infected adult patients (at least 18 years old) enrolled at Felege Hiwot Teaching and Specialized Hospital, Amhara region, northwest Ethiopia. The hospital started the free HAART program in 2005 when there was limited ART service in public health institutions. However, the data consists of a record of patients with at least two follow-up visits as from September 2008 to August 2012 of the study period. Before starting HAART, patients were given health and HAART related education. Patients visited the Teaching and Specialized Hospital monthly for the first six months for HAART and thereafter quarterly for the remaining study period to get HAART medication for the subsequent months and for the review of the progression of their CD4 cell count change. An administrative permission was given by respective ethical committee of two universities namely Bahir Dar University Ethical approval committee (which belongs to Bahir Dar University), Ethiopia with Ref≠ RCS/1412/2006 and School of Science Research Ethics Review Committee (which belongs to University of South Africa, South Africa, Ref#2015 − *SSR* − *ERC*_006, to use secondary data for current research. We can attach the written ethical statements up on request. The quality of data was controlled by the ART section of the hospital.

### Response variables in the data

The response variable of interest, CD4 cell count change, was calculated from the laboratory determined CD4 cell counts of the patients at follow-up visits as:

1 $$ \boldsymbol{\Delta} {\mathrm{y}}_{\mathrm{ij}}={\mathrm{y}}_{\mathrm{ij}}-{\mathrm{y}}_{\mathrm{ij}-1} $$

where y_ij_ and y_ij − 1_are the respective measured CD4 cell counts of patient i at the j^th^ and (j-1)^th^ follow-up visits. The other response variable of interest was the time to default from HAART or equivalently the number of follow-up visits to default from HAART.

### Predictor variables in the data

The time invariant predictors were: sex (Male, Female); residential area (Urban, Rural); level of education (No education, Primary, Secondary and Tertiary); marital status (Living with partner, Living without partner); level of income (Low, Middle and High); WHO stages of HIV (Stage1, Stage2, Stage3 and Stage4); ownership of cell phone (Yes, No), whether or not the patient disclosed the disease (Yes, No); age in years; and baseline CD4 cell count in cells/mm3. Descriptive statistics of these variables are in the RESULTS section of this paper.

### The generalized linear mixed effects model

Let *y*_*ij*_ (*i = 1,2,…,n*; *j = 1,2,…*,*n*_*i*_) be the CD4 count change of patient *i* at follow up visit time *j*; ***Y***_***i***_ = ${\left({y}_{i1},{y}_{i2},\dots .{y}_{i{n}_i}\right)}^T;{\boldsymbol{X}}_{i1}^T$ be an *n*_*i*_ × *p* design matrix of fixed effects for patient *i* that is associated with the *p*-dimensional vector ***β***_**1**_ of fixed effects; and ${\boldsymbol{Z}}_{i1}^T$ be an *n*_*i*_ × *q* design matrix of random effects for patient *i* that is associated with the *q*-dimensional vector ***ν***_***i***_ of patient specific random effects. Then, if the conditional distribution of ***Y***_***i***_ given ***ν***_***i***_ is from the exponential family, the generalized linear mixed effects model for ***Y***_***i***_ can be written as:

2 $$ g\left({\boldsymbol{Y}}_{\boldsymbol{i}}\right)={{\boldsymbol{X}}_{i1}}^T{\boldsymbol{\beta}}_1+{\boldsymbol{Z}}_{i1}^T{\boldsymbol{\nu}}_{\boldsymbol{i}}+{\boldsymbol{\varepsilon}}_i $$

where g(.) is the link function that is completely specified by specifying the conditional of ***Y***_***i***_ in the exponential family. For example, g (.) = *log* (.) if the distribution is Poisson as is possibly the case with the CD4 cell count change. Usually, ***ν***_***i***_ is assumed to be multivariate normally distributed with mean vector zero and covariance matrix, ***ν***_***i***_, and ***ε***_***i***_ assumed to be multivariate normally distributed with mean vector zero and covariance matrix ***I***${\sigma}_{\varepsilon}^2.$ The term ${\boldsymbol{Z}}_{i1}^T{\boldsymbol{\nu}}_{\boldsymbol{i}}$ in model (2) accounts for patient level variation in CD4 cell count change. Model (2) was fitted to the data using Proc glimmix in SAS Version 9.2.

### Survival time models

Let *t*_*j*_ (*j = 1,2,…,n*) be the time to default from HAART for patient *i*; and ${\boldsymbol{X}}_{i2}^T$= (*x*_12_, *x*_22_, …*x*_*k*2_) be a *p*-dimensional fixed effects vector of covariates for patient *i* that is associated with the *p*-dimensional vector ***β***_**2**_ of fixed effects. Then both the parametric and the semi parametric models for *t*_*j*_ have hazard functions at the time *t* of the form:

3 $$ {h}_i(t)={h}_0\;(t)\;\mathit{\exp}\;\left[{{\boldsymbol{X}}_{i2}}^T{\boldsymbol{\beta}}_2\right] $$

with the difference that parametric models specify the baseline hazard function *h*_0_(*t*) (or equivalently the distribution of *t*_*j*_). The usual specified distributions include the Weibull, the Exponential, the Log logistic and the Log normal. For example, when the distribution is Weibull model (2) becomes the parametric model:

4 $$ {h}_i(t)=\varnothing \rho {t}^{\rho -1}\;\mathit{\exp}\;\left[{{\boldsymbol{X}}_{i2}}^T{\boldsymbol{\beta}}_2\right] $$

where ∅ is the dispersion parameter and *ρ* is the shape parameter of the distribution. The semi-parametric model (unspecified to *h*_0_ (*t*)) is the widely used Cox Proportional Hazards (PH) model [17]. The parametric and semi-parametric models were fitted to the data using Proc glimmix in SAS Version 9.2.

In this study, the direct formulation of joint modeling of both CD4 cell count change and time to default from HAART with the introduction of Bayesian perspective within Markov Chain Monte Carlo (MCMC) structures [18] was adopted. The generalized linear mixed model for CD4 cell count change becomes:

5 $$ g\left({\boldsymbol{Y}}_{\boldsymbol{i}}\right)={{\boldsymbol{X}}_{i1}}^T{\boldsymbol{\beta}}_1+{\boldsymbol{Z}}_{i1}^T{\boldsymbol{\nu}}_{\boldsymbol{i}}+{\boldsymbol{\varepsilon}}_i $$

as in model (2) but with the assumption that ***ε***_*i*_~ *N*(**0**, *b*_*i*_*I*) and log(*b*_*i*_)~ *N*(0, ${\sigma}_b^2$) [19]. Here, *b*_*i*_ denotes for the actual variability for specific- patients which follows a log-normal distribution with mean 0 and variance ${\sigma}_b^2$ [20].

The corresponding survival time model is expressed as:

$$ {h}_i(t)={h}_0(t)\;\mathit{\exp}\;\left[{{\boldsymbol{X}}_{i2}}^T{\boldsymbol{\beta}}_2\right]+{W}_{\boldsymbol{i}2} $$

where ${W}_{\boldsymbol{i}\mathbf{2}}={\boldsymbol{\nu}}_{\boldsymbol{i}}^{\boldsymbol{T}}\boldsymbol{\tau} +{\tau}_{q+1}\log {b}_i+\kern0.5em {\nu}_{q+1}$ (with ***ν***_***i***_ and *b*_*i*_ as defined in model (5), (***τ***^*T*^***,*** *τ*_*q* + 1_) a vector of parameters, and  *ν*_*q* + 1_~ *N*(0, ${\sigma}_{\nu}^2$), a frailty effect is an added heterogeneity term to model (3) in order to account for communal and patient specific random effects. In this context model (4) becomes:

6 $$ {h}_{\boldsymbol{i}}(t)=\varnothing \rho {t}^{\boldsymbol{\rho} -1}\mathit{\exp}\kern0.24em \left[\ {\boldsymbol{X}}_{i2}^T{\boldsymbol{\beta}}_2\right]+{W}_{i2} $$

Thus, the models for CD4 cell count change and time to default from HAART are correlated through the random effect models:

7 $$ {W}_{i1}={\boldsymbol{Z}}_{i1}^T{\boldsymbol{\nu}}_{\boldsymbol{i}} $$

and

8 $$ {W}_{\boldsymbol{i}\mathbf{2}}={\boldsymbol{\nu}}_{\boldsymbol{i}}^{\boldsymbol{T}}\boldsymbol{\tau} +{\tau}_{q+1}\log {b}_i+\kern0.5em {\nu}_{iq+1} $$

In this study, **ν**_i_ = (ν_i0_)T is the random of patient i’s effect and  ν_02_ is the random fraility term.

### Joint model selection

The specific nature of random effect models (7) and (8) is selected using the Deviance Information Criterion (DIC) [15, 21] which is a hierarchical form of common Akaike Information Criteria (AIC) [22]. As with the AIC, a model with the smallest DIC is preferred.

## Results

The data was analyzed using SAS Version 9.2. Table 1 displays the summary statistics of the predictor variables in the data. Among the sample of 792 patients: 40.9% were rural residents; 50.6% were females; 44.8% were living with their partner; 72.6% of them disclosed their disease; 50.5% were owners of cell phone; and 68.2% of the patients had good adherence to HAART.

**Table 1:** Descriptive statistics of potential predictor variables of CD4 cell count change and time to default from HAART in the data sample size 792

Variable		Average	No (%)
Weight (kg)		62 (58, 70)	–
Baseline CD4 count cells/ mm^3^		150 (113, 198)	–
Age (years)		36 (28, 48)	–
First month / initial CD4 cell count change/mm^3^		15.9 (12-26)	–
Sex	Male		391 (49.4)
	Female		401 (50.6)
Educational status	no education		160 (20.2)
	Primary		205 (25.9)
	Secondary		273 (34.5)
	Tertiary		154 (19.4)
Residential area	Urban		468 (59.1)
	Rural		324 (40.9)
Marital status	Living with partner		355 (44.8)
	Living without Partner		437 (55.2)
Level of Income	Low income (< 500 ETB per month)		355 (44.8)
	Middle income (5001-999 ETB per month)		346 (43.7)
	High income (≥1000ETB per month)		91 (11.5)
WHO HIV Stage	Stage I		101 (12.8)
	Stage II		258 (32.6)
	Stage III		199 (25.1)
	Stage IV		234 (29.5)
Disclosure	Yes		575 (72.6)
	No		217 (27.4)
Cell ownership	Yes		400 (50.5)
	No		392 (49.5)
First month HAART adherence	Good		540 (68.2)
	Fair		160 (20.2)
	Poor		92 (11.6)

A decision had to be made on whether or not the conditional distribution of the CD4 cell count change was standard Poisson, quasi-Poisson or negative Binomial.

**Fig. 1:**
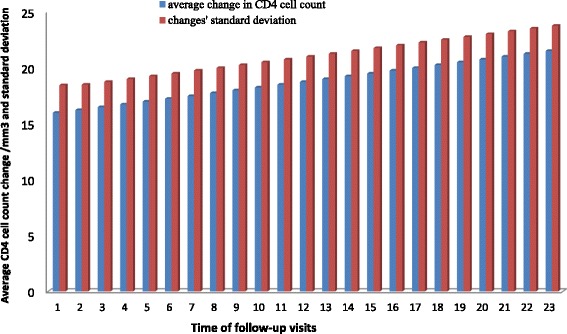
The average CD4 cell count change versus the corresponding standard deviation at each follow-up visits

Figure 1 displays the graph of the average CD4 cell count change versus the corresponding standard deviation at each follow-up visit time. The graph shows that at each visiting time, the variance was greater than the mean which suggested that the distribution of the CD4 cell count change was over dispersed, and hence could be either be negative Binomial or quasi-Poisson. The quasi-Poisson model was preferred, because the average CD4 cell count change was greater than the cut-off point [14], and because the comparison of the negative Binomial and the quasi-Poisson model using the information criteria statistics (the smaller is the better) in Table 2 favored the quasi-Poisson model.

**Table 2:** Comparison of quasi-Poisson and negative Binomial models using information criteria

Criterion	quasi-Poisson			negative-Binomial		
	Value	d.f	Value/d.f	Value	d.f	Value/d.f
Pearson Chi-square	1309	773	1.693	1530	773	1.98
Log likelihood	− 2159			− 2658		
AIC	4355			5354		
BIC	4444			5443		

To fit the survival model, the parametric (Weibul models) and semi-parametric (Cox proportional hazard Model) models were take in to consideration and the two models were compared using AIC. As usual, the goodness of fit of the Weibul and Cox Proportional models were compared using a test statistics such as the Akakai information criteria (AIC) and Bayesian information criteria (BIC) and Pearson Chi-square divided by degree of freedom, assuming the smaller value as the better one. The comparison between survival models (Weibul and Cox proportional models) is shown in Table 3.

**Table 3:** Comparison of Weibul and Cox regression models using information criteria

Criterion	Weibul model			Cox regression model		
	Value	d.f	Value/d.f	Value	d.f	Value/d.f
Pearson Chi-square	1409	773	1.83	1630	773	2.11
Log likelihood	− 2059			−2658		
AIC	4255			5454		
BIC	4544			5543		

Table 3 indicates that Weibul has smaller Pearson chi-square/d.f, AIC and BIC. Hence Weibul was in favor of Cox proportional hazards model and it fitted the data well as compared to proportional hazards model.

Models (2) and (5) were fitted to the data using Proc glimmix in SAS Version 9.2. The two models are shown in Table 4, and are not very different from each. However, model (5) fitted the data better because model (5) has smaller posterior estimates for many of the predictors.

**Table 4:** Posterior means and correspondence *p*-values for parameter estimation with inclusion and exclusion of patient specific variance

Effect	Model (2): With homogeneity of variance for patients		Model (5): With patient specific variance/heterogeneity	
	Posterior estimate	*p*-value	Posterior estimate	*p*-value
Intercept	4.350	0.004	4.130	0.002
Age	−0.006	0.003	− 0.036	0.005
Weight	0.001	0.082	0.001	0.082
Baseline CD4	0.005	0.008	0.002	0.008
Area (Reference = Urban)				
Rural	−0.002	0.064	−0.003	0.003
Marital Status (Reference = without partner)				
With partner	0.007	0.003	0.005	0.003
Sex (Reference = male)				
Female	0.034	0.004	0.023	0.003
Level of education (Reference = Tertiary)				
No educationl	0.001	0.076	0.001	0.076
Primary education	0.017	0.044	0.017	0.034
Secondary education	0.020	0.028	0.010	0.038
Level of Income (Reference = High income)				
Low income	−0.010	0.074	−0.020	0.064
Middle income	−0.006	0.062	−0.016	0.052
Owner of Cell phone (Reference = With phone)				
Without cell phone	−0.001	0.004	−0.001	0.004
Level of adherence (Reference = Good adherence)				
Poor adhere	−0.523	0.004	−0.483	0.002
Fair adhere	−0.452	0.001	−0.552	0.002
Level of disclosure the disease (Reference = yes)				
No	- 0.00195	0.0031	−0.1020	0.0031
WHO stages (Reference = Stage IV)				
Stage I	0.1511	0.0021	0.2511	0.0021
Stage II	0.1567	0.0321	0.1567	0.0321
Stage III	0.1381	0.2311	0.4381	0.0311
Time	0.0210	0.0013	0.1210	0.0213
Var (ν_i0_)	0.8820		0.8620	
Var (ν_*i*1_)	0.0210		0.0110	
Cov (ν_i0_, ν_i1_)	- 0.0542		- 0.0642	
Corr (ν_i0_, ν_i1_)	−0.8214		−0.7314	
DIC	234,763		212,393	

ν_i0_ is the random of patient i effect and  ν_i1_ is the random fraility term

Estimation of parameter in both the full Weibull and exponential models were similar to each other, but the estimated Weibull shape parameter, *ρ* was 0.763 with 95% CI (0.484, 0.990) which is less than 1 and indicates that default rate decreased as the number of follow-ups increased.

In view of the fact that results and conclusions of this study will be most valid and reliable if the missing observations (due to patient dropouts/defaulters) are missing completely at random (MCAR), the data was analyzed as described in Table 4 to assess the data missing mechanism. A logistic regression model with 0 = missing and 1 = not missing responses was fitted to the data to obtain Table 5. All the effects are insignificant which suggests that MCAR was the data missing mechanism.

**Table 5:** Posterior effects of the predictor variables from modeling CD4 cell count change with models (2) (no patient specific variance) and (5) (with patient specific variance)

Parameters	Estimate	Standard error	*P*-values
Intercept	1.808	0.910	0.470
Age	− 0.658	0.145	0.831
Follow up times	0.047	0.052	0.058
Previous CD4 cell count (ref=*y*_*j*_ ≤ *y*_*j* − 1_) or *I*_*i*_ = 0			
*I*_*i*_ = 1 (*y*_*j*_ > *y*_*j* − 1_)	0.072	0.320	0.650
Gender (ref = female)			
Male	0.003	0.084	0.976
Residence area (ref = Urban)			
Rural	−0.380	0.083	0.648
Marital status(ref = living without partner)			
Living with partner	0.109	0.086	0.208

where *y*_*j*_  is the CD4 cell count at the j^th^ follow-up visit; *y*_*j* − 1_ is the CD4 cell count at the (j-1)^th^ follow-up visit

### Joint model results

First the separate data analyses were conducted and then two joint models with different latent process were conducted for the fluctuation of repetitive CD4 cell count. The first were joint models with homogeneous variance assumptions using (1) and the other was joint models with heterogeneous variance of CD4 count change for each individual (5). Hence, the longitudinal sub model was described by both the common/conventional generalized linear mixed effects model and by the generalized linear mixed effects model including patient-explicit variances. Alternatively, the time to default from the HAART sub-model was fitted using a full Weibul distribution and the two sub-models were related applying communal covariates.

Based on the baseline variables, the longitudinal sub models were constructed using the usual generalized linear mixed effect model with the assumption of homogeneous patient specific CD4 cell count change and log likelihood function was reduced as it was done in the joint models with inclusion of different random effects and different forms of latent processes *W*_1_ (t) and *W*_2_ (t). In constructing joint models, the simple joint model (model, (2)) with no random effect in the two sub model was conducted. Next, joint models with random intercept *v*_0_ and a frailty term *v*_3_ were constructed successively. The inclusion of a frailty term, *v*_3_ in the time to default sub-model, leads for improvement of the model. Hence, DIC decreased as frailty terms included in the time-to default sub model *W*_2_(t).

The correlation between *W*_1_(t) and *W*_2_(t) was introduced using communal random intercept, *v*_0_ and this indicates that the DIC further decreased. In addition to the random intercept, the random slope was also included in the longitudinal data analysis. The inclusion of random slope also reduced the DIC. The result of this subsequent reduction of DIC is indicated in Table 6.

**Table 6:** Model selection for joint data analysis using generalized linear mixed effect for longitudinal data and Weibul Survival models for survival data

Random Effects	W_1_(t)	W_2_(t)	DIC
Only fixed effect	0	0	3547
Fixed effect +random intercepts only	*v*_0_	0	3506
Fixed effect +Random intercept+ random intercepts and slopes	*v*_0_+*v*_1_(t)	*τ*_0_*v*_0_+*τ*_1_*v*_1_+*v*_3_	3481

Table 6 indicates that the inclusion of random intercept as well as random intercept and slopes substantially reduced the DIC.

On the other hand**,** the generalized linear mixed effect model with the inclusion of subject-explicit CD4 cell count change variability in longitudinal data analysis was conducted. These joint models relate the variability existed in CD4 cell count changes to the follow-up visits defaulting from HAART. This relation shows that fluctuation(ups and downs) of the longitudinal outcome (CD4 cell count change) can be quantified as hazard ratio [23]. Hence, the inclusion of patient/subject specific CD4 cell count fluctuation in joint model improves the model to fit the data well. The selected model indicates that patient’s survival was related to the increase of rate of CD4 cell count and decrease of its fluctuation/variability of CD4 cell count which further indicates that the increase of CD4 count change for a particular patient leads to better health status and such patient has less likely in defaulting from treatment. While, patients with high variability of CD4 cell count change leads to poor health conditions and has more likelihood in defaulting from HAART.

### Separate and joint model comparisons

Considering the final selected joint model, the results obtained from this model was compared to the separated models (without latent association introduced by W_2_). The models with subject specific CD4 cell count change variability had smaller DIC as compared to models without subject specific CD4 cell count variability. To compare the joint and separate models, first subject specific CD4 cell count change incorporated the variability to both models were considered. The comparison of the two approaches was indicated in Table 7. During comparison, only significant predictors at separate models were considered.

**Table 7:** A separate and joint model comparison for longitudinal CD4 cell count change and time to default from HAART

parameter	Separate models		Joint models	
	Posterior mean	*p*-value	Posterior mean	*p*-value
Longitudinal sub models				
Intercept	4.350	0.004	4.130	0.002
Age	−0.006	0.003	− 0.036	0.005
Weight	0.001	0.082	0.001	0.082
Baseline CD4	0.004	0.008	0.002	0.008
Marital Status (Reference = without partner)				
With partner	0.007	0.003	0.005	0.003
Sex (Reference = male)				
Female	0.034	0.004	0.023	0.003
Ownership of cell phone (Reference = With phone)				
Without cell phone	−0.001	0.004	−0.001	0.004
Level of adherence (Reference = Good adherence)				
Poor adherence	− 0.523	0.004	− 0.483	0.002
Fair adherence	−0.452	0.001	−0.552	0.002
Level of disclosure of the disease (Reference = yes)				
No	−0.002	0.003	−0.102	0.003
WHO stages (Reference = Stage IV)				
Stage I	0.151	0.002	0.251	0.002
Stage II	0.1567	0.032	0.157	0.032
Stage III	0.1381	0.231	0.438	0.031
Time	0.021	0.001	0.121	0.021
Var (*V*_0*i*_)	0.882	0.032	0.862	0.023
Var (*V*_1*i*_)	0.021	0.043	0.011	0.005
Cov (*V*_0*i*_, *V*_1*i*_)	- 0.054	0.032	- 0.064	0.006
Corr (*V*_0*i*_, *V*_1*i*_)	−0.821	0.021	−0.731	0.001
Survival sub models				
Intercept	1.202	0.003	1.402	0.004
Age	−0.006	0.003	−0.036	0.005
Baseline CD4	0.004	0.008	0.002	0.008
Marital Status (Reference = without partner)				
With partner	0.007	0.003	0.005	0.003
Sex (Reference = male)				
Female	0.034	0.004	0.023	0.003
Without cell phone	−0.001	0.004	− 0.001	0.004
Level of adherence (Reference = Good adherence)				
Poor adherence	− 0.523	0.004	− 0.483	0.002
Fair adherence	−0.452	0.001	−0.552	0.002
Level of disclosure the disease (Reference = yes)				
No	− 0.002	0.003	− 0.102	0.003
WHO stages (Reference = Stage IV)				
Stage I	0.151	0.002	0.251	0.002
Stage II	0.157	0.032	0.157	0.032
Stage III	0.138	0.231	0.438	0.031
Time(visiting time)	0.021	0.001	0.121	0.021
*τ*_1_			−2.324	0.005
*τ*_3_			0.051	0.003
*ρ*	0.654	0.004	0.864	0.003

Table 7 indicates that, the posterior estimates of the correlated parameters at joint model analysis were considerably different from zero and this is an indication of the correlation between the two sub models. The estimate of the shape parameter in the CD4 cell count fluctuation is negative (*τ*_1_= − 2.324) which indicates that the increase in the fluctuation of the CD4 cell count change was negatively associated with the number of follow-up visits in HAART. The positive value of association for parameters in the CD4 cell count change variability indicates that there is a direct proportionality between the CD4 cell count change fluctuation and the hazard of defaulting from HAART. Hence, as the CD4 cell count change variability increased, the hazard of defaulting also increased.

The convergence of the final joint models was checked with time series of iterations. There was a higher degree of randomness between successive iterations and this indicates that the value converged to a particular target density. The estimated hazard ratio and 95% credible intervals for the joint survival and CD4 cell count change data were indicated in Table 8.

**Table 8:** Average fluctuation of CD4 cell count and Hazard Ratio estimates for final selected joint models

parameter	Parameter estimate		Hazard Ratio (HR) estimate.	
	Average fluctuation	95% Credible interval	HR	95% Credible intervals
Intercept	−4.350	(−7.435, −2.856)	0.1295	(0.082, 0.454)
Age	−0.006	(−0.003, −0.019)	0.0362	(0.009, 0.052)
Weight	0.001	(−0.008, 0.002)	0.0056	(0.001,0.008)
Baseline CD4 count	0.004	(0.001, 0.025)	0.0015	(0.001,0.002)
Marital Status (Reference = without partner)				
With partner	0.007	(0.003, 0.009)	0.005	(0.003, 0.008)
Sex (Reference = male)				
Female	0.034	(0.004,0.067)	0.023	(0.003,0.075)
Ownership of cell phone (Reference = With phone)				
Without cell phone	−0.006	(−0.004, − 0.024)	0.007	(0.004, 0.009)
Level of adherence (Reference = Good adherence)				
Poor adherence	−0.523	(− 0.644, − 0.235)	0.483	(0.002, 0.621)
Fair adherence	−0.452	(− 0.671, − 0.253)	0.552	(0.092,0.831)
Level of disclosure of the disease (Reference = yes)				
No	−0.002	(− 0.073, − 0.001)	0.1020	(0.003,0.324)
WHO stages (Reference = Stage IV)				
Stage I	0.151	(− 0.002, 0.246)	0.2511	(0.081, 0.452)
Stage II	0.157	(0.032, 0.224)	0.1567	(0.092, 0.421)
Stage III	0.138	(−0.231, 0.231)	0.4381	(0.231, 0.643)
Time	0.021	(0.001, 0.064)	0.1209	(0.091, 0.241)
*τ*_1_	−2.965	(−4.547, −1.234)	0.0341	(0.010, 0.067)
*τ*_3_	0.653	(0.254, 0.923)	1.7342	(1.234, 1.966)

As presented in Table 8, some covariates such as age, baseline CD4 cell count, marital status, cell phone ownership, adherence level, disclosure’s level of disease and the number of follow-ups of patients had significant effect on both the repetitive measures of the CD4 cell count fluctuation and the number of followed-up visits required to default from HAART. Hence, as age of a patient increased, the CD4 cell count change as well as its waiting time in the HAART decreased. However, whenever a patient started his/her HAART with a relatively high baseline CD4 cell count, the number of CD4 cell count and waiting time in the HAART also increased. Patients without the ownership of cell phone had decreased by 0.01 (*e*^−0.006^) his/her improvement of CD4 cell count change as compared to patients who owned cell phone. Fair adherent patients had 33% less probability to have improvement in their CD4 cell count as compared to good adherent patients. Similarly, poor adherent patients had 41% less likelihood to have improved their CD4 cell count as compared to good adherent patients. Such poor adherent patients had a short waiting time in the HAART. As the visiting times/ the number of follow-ups visits of a patient increased by one unit, the improvement of his/her CD4 cell count increased by 2.1%, keeping the other variables constant. Female patients had 3.4% improvement in their CD4 cell count change as compared to males.

## Discussions

The study was conducted using a Bayesian approach to jointly model the CD4 cell count change and the time to default from HAART. The results of the current study indicate that the joint model was in favor of separate models for determining the predictors of CD4 cell count change and time to default. This outcome is complemented by the results of previous research [15] . Another study stated that joint models showed a significant difference between treatment groups that was not identified by the separate model data analyses [18]. Since the correlated parameters in the communal random effect models measure the link between the two sub models, the associated information or common predictors of the repetitive outcomes and the time to default from HAART can be identified easily. The joint model had a smaller posterior mean as compared to the separate models and this indicates that the joint model fits the data well as compared to the separate models. The inclusion of subject specific variability in longitudinal CD4 cell count change improved the model significantly. Elder patients had higher CD4 cell count change fluctuation as compared to youngsters and this indicates that elders had low CD4 cell count change improvement and had short waiting time in HAART. This might be related to the case that elders are non-adherent as compared to youngsters [24]. On the other hand, it is known that as patient’s age increased, his/her CD4 cell count decreased and such a patient might be defaulted because of death and other reasons. Previous research indicates that CD4 cell count change had been affected by sex, clinical stages and educational levels [15]. Females had a better CD4 cell change improvement as compared to males; this might be the reason that females have good experience in prenatal and postnatal healthcare follow-ups as compared to males. Females also had experience in taking pills for family planning. This experience made female patients to be adherent to the prescribed medication properly and to have long waiting time in HAART program as compared to males. As the number of follow-ups for patients increased, the defaulting rate of patients from the HAART program decreased. This might be happen since patients with long visiting time acquire more experience to adhere to the medication and this leads to improve their CD4 cell count progression and such patients might have long waiting time (greater number of follow-ups) in the HAART program. Patients with ownership of cell phone had a better CD4 cell count progress (growth) as compared to those without cell phone. Patients who used cell phone as memory aid had a better CD4 cell count progress/low CD4 cell count fluctuation. This memory aid helps to take pills on time and this has its own impact on the progress of the CD4 cell count and on the longevity of patients in the HAART program.

## Conclusion

Certain groups that require intervention had been identified in the current research and these groups need special attention for the longevity of their life within HAART. An integrated intervention becomes effective for the patients to survive for long period of time with in the treatment. Ministry of Health or health staff should advise the patients to adhere to the prescribed medication properly to improve their CD4 cell count change/progress and to have a long time to default from HAART program. Health education should be given to patients who are living in rural areas, and who came after a declined number of CD4 cell count for diagnosis. Medical advice should also been given to patients to disclose the disease to get social support from families and communities and to have longer waiting time within the treatment.

The current research had limitations that the results obtained using a Bayesian approach may be different if likelihood approaches would be included and triangulated. This gap might be considered as potential area for future researchers. The data had been taken from one treatment site. If such data would be collected from different health institutions, the results may be different. The results of the current investigation are useful to make integrated intervention in providing health education to the patients, and would help to guide the policy and management of HAART. Further studies are recommended with additional predictors such as nutrition, religion, and consumption of substances on CD4 cell count change. The quality of health service provision which is not included under this study may have direct or indirect effects on patients’ CD4 cell count change and this also needs further investigation in for future researches.

## Abbreviations

CD4: Classification Determinant Four
DIC: Deviance information criteria
GLMM: Generalized Linear mixed model
HAART: Highly Active Ant-Retroviral Therapy
HIV: Human Immune Deficiency Virus
MCAR: Missed completely at random
OR: Odds Ratio
WHO: World Health Organization
